# Birth Outcomes of Neonates Exposed to Marijuana in Utero: A Systematic Review and Meta-analysis

**DOI:** 10.1001/jamanetworkopen.2021.45653

**Published:** 2022-01-27

**Authors:** Greg Marchand, Ahmed Taher Masoud, Malini Govindan, Kelly Ware, Alexa King, Stacy Ruther, Giovanna Brazil, Hollie Ulibarri, Julia Parise, Amanda Arroyo, Catherine Coriell, Sydnee Goetz, Amitis Karrys, Katelyn Sainz

**Affiliations:** 1 Marchand Institute for Minimally Invasive Surgery, Mesa, Arizona; 2 Faculty of Medicine, Fayoum University, Fayoum, Egypt; 3 International University of the Health Sciences, Basseterre, Saint Kitts; 4 Midwestern University College of Osteopathic Medicine, Glendale, Arizona; 5 Department of Pediatrics, Tucson Medical Center, Tucson, Arizona

## Abstract

**Importance:**

While some studies have found an association between marijuana use and adverse neonatal outcomes, results have not been consistent across all trials.

**Objective:**

To assess available data on neonatal outcomes in marijuana-exposed pregnancies.

**Data Sources:**

PubMed, Medline, ClinicalTrials.gov, Cochrane, Scopus, and Web of Science were searched from each database's inception until August 16, 2021.

**Study Selection:**

All interventional and observational studies that included pregnant women who were exposed to marijuana compared with pregnant women who were not exposed to marijuana and that reported neonatal outcomes were included.

**Data Extraction and Synthesis:**

Reporting followed the Preferred Reporting Items for Systematic Reviews and Meta-analyses guideline. Data were extracted by 2 authors for all outcomes, which were pooled using a random-effects model as mean difference or risk ratio (RR) and 95% CI. Data were analyzed from August through September 2021.

**Main Outcomes and Measures:**

All outcomes were formulated prior to data collection. Outcomes included incidence of birth weight less than 2500 g, small for gestational age (defined as less than the fifth percentile fetal weight for gestational age), rate of preterm delivery (defined as before 37 weeks’ gestation), gestational age at time of delivery, birth weight, incidence of neonatal intensive care unit (NICU) admission, Apgar score at 1 minute, Apgar score at 5 minutes, incidence of an Apgar score less than 7 at 5 minutes, fetal head circumference, and fetal length.

**Results:**

Among 16 studies including 59 138 patients, there were significant increases in 7 adverse neonatal outcomes among women who were exposed to marijuana during pregnancy vs those who were not exposed during pregnancy. These included increased risk of birth weight less than 2500 g (RR, 2.06 [95% CI, 1.25 to 3.42]; *P* = .005), small for gestational age (RR, 1.61 [95% CI, 1.44 to 1.79]; *P* < .001), preterm delivery (RR, 1.28 [95% CI, 1.16 to 1.42]; *P* < .001), and NICU admission (RR, 1.38 [95% CI, 1.18 to 1.62]; *P* < .001), along with decreased mean birth weight (mean difference, −112.30 [95% CI, −167.19 to −57.41] g; *P* < .001), Apgar score at 1 minute (mean difference, −0.26 [95% CI, −0.43 to −0.09]; *P* = .002), and infant head circumference (mean difference, −0.34 [95% CI, −0.63 to −0.06] cm; *P* = .02).

**Conclusions and Relevance:**

This study found that women exposed to marijuana in pregnancy were at a significantly increased risk of some adverse neonatal outcomes. These findings suggest that increasing awareness about these risks may be associated with improved outcomes.

## Key Points

**Question:**

Are adverse neonatal outcomes associated with exposure to marijuana among mothers during pregnancy?

**Findings:**

In this meta-analysis of 16 studies including 59 138 patients, maternal marijuana exposure during pregnancy was associated with increased risk of preterm deliveries and neonatal intensive care unit admission and decreased mean birth weight, 1-minute Apgar score, and head circumference of babies. There were no differences in mean 5-minute Apgar score or total infant length.

**Meaning:**

This study found that women using marijuana during pregnancy may be at increased risk of some adverse neonatal outcomes.

## Teaser

This systematic review and meta-analysis compares adverse neonatal outcomes among mothers exposed to marijuana during pregnancy vs mothers not exposed to marijuana during pregnancy in 16 cohort studies.

## Introduction

Misuse of marijuana (the drug is generally referred to as marijuana for the smoked or ingested substance and cannabis for plant parts or derivatives) is one of the most prevalent substance use disorders, particularly among young adults, and the demands for worldwide treatment have increased.^1^ Marijuana (*Cannabis sativa* L) belongs to the Cannabaceae family and is grown extensively globally.^2^ During pregnancy, self-reported use of marijuana overall has ranged from 2% to 5% in several studies.^3^ However, some studies have reported that when limited to populations of young women living in urban areas who are less advantaged socioeconomically, that number could be as high 15% to 28%.^3^ Singh et al^4^ reported that the prevalence of prenatal cannabis use was as high as 22.6% among their included studies from different countries. Authors report that testing for marijuana use at the time of delivery is associated with increased rates of use than is self-reported during prenatal care.^5^ This finding, in part, may be secondary to the fact that many mothers using marijuana during pregnancy may not seek prenatal care at all.^6^ Some authors^7^ have suggested that the prevalence is thus likely underestimated, given that marijuana use is often underreported.

The prevalence of marijuana use during pregnancy may continue to increase, given that there is a suggested association between legalized recreational marijuana and increased use in prenatal and postpartum periods.^8,9^ Remarkably, 34% to 60% of individuals who use marijuana keep using it during pregnancy.^10^ Many women cite the belief that marijuana use is relatively safe during pregnancy among other reasons for continuing use.^10,11,12,13^

Cannabis products may be associated with changes in fetal biology, given that the Δ9-tetrahydro-cannabinol crosses the placenta and can be identified in the adult body for 30 days.^14,15,16^ Cannabinoid receptors are present in the central nervous system of a developing fetus at the beginning of the second trimester.^17^ Exposure to exogenous cannabinoids may be associated with changes in the prefrontal cortex and theoretically with its development and function.^18^

Several studies^19,20^ have found an association between marijuana use and adverse neonatal outcomes, including small for gestational age, low birth weight, preterm birth, stillbirth, and maternal hypertensive disorders. These findings have not been consistent across all studies.^21^ There have been mixed results for the association between maternal marijuana use and infant birth weight in previous reviews and meta-analyses assessing marijuana use during pregnancy.^7,19,22^ We sought to perform the largest meta-analysis to date, to our knowledge, on all available high-quality data to investigate the association of marijuana use during pregnancy with neonatal outcomes.

## Methods

This study is a systematic review and meta-analysis and follows the Preferred Reporting Items for Systematic Reviews and Meta-analyses (PRISMA) reporting guideline.

### Literature Search

An electronic search was performed on PubMed, Medline, ClinicalTrials.gov, Cochrane, Scopus, and Web of Science from their inception until August 16, 2021, for related records. The used search strategy included the following: *smoking*, *marijuana*-*marihuana smoking*-*smoking*, *marihuana*-*smoking*, *blunts*-*blunts smoking*-*blunts smokings*-*smokings*, *blunts*-*smoking blunts*-*blunt*, *smoking*-*blunts*, *smoking*-*smoking blunt*-*hashish smoking*-*smoking*, *hashish*-*cannabis smoking*-*smoking*, *cannabis infants*, and *newborn*-*newborn infant*-*newborn infants*-*newborns*-*newborn*-*neonate*-*neonates*.

### Inclusion and Selection Criteria

Inclusion criteria included interventional and observational (ie, case-control, cohort, and cross-sectional) studies that included pregnant women exposed to marijuana compared with pregnant women who were not exposed and that reported any of our selected neonatal outcomes. Exclusion criteria included studies that were not interventional or observational, case studies and letters to editors, studies that did not include any of our selected outcomes, and non–English language abstracts. We removed duplicates using EndNote software version 8 (Clarivate Analytics). Then, we screened titles and abstracts, followed by full-text screening to identify relevant studies. Screening was performed independently by 2 authors (G.M. and A.T.M.); a third author (G.B.) was used for any disagreement until consensus was reached. In addition to identifying studies by our search strategy, we also screened references from our synthesized studies to be sure no additional qualifying studies were missed.

### Quality Assessment

To appropriately assess the quality of the 16 observational cohort studies included in our synthesis, we performed a full quality assessment. This assessment was undertaken according to a tool from the National Institute of Health (NIH) Study Quality Assessment Tools.^23^ This tool includes 14 questions to grade study quality with a final score out of 14. The questions included judgments regarding the clarity of the study question, definition of the study population, participation rate, prespecification of the study inclusion and exclusion criteria, sample size justification, outcome measurement process, sufficiency of the time frame and follow-up period, precise definition and validity of the exposure and outcome measures, multiple measurements of the exposure, blinding of the outcome assessor, loss of follow-up rate, and potential confounding variables. The answers were yes, no, not applicable, cannot determine, or not reported. Quality judgments were made by 2 different authors (G.M. and K.S.), and any disagreement was resolved by consensus or by a third author (A.T.M.) if necessary. Studies were given an overall score according to which their quality was judged as good, fair, or poor.

### Data Extraction

We extracted data related to the following: (1) summary of included studies, including study design, country, study arms and sample, marijuana exposure details, and results; (2) baseline characteristics, including study group, sample size, maternal age (in years), parity, and alcohol use; and (3) study outcomes, including neonatal outcomes. Author consensus was used to decide which studies were eligible for each synthesis.

### Study Outcomes

Outcomes of interest were determined by the authors prior to the collection of data for this study. The outcomes included the rate of babies born at low birth weight (defined as all births <2500 g), small for gestational age (defined as a weight less than the fifth percentile at birth), rate of preterm delivery (<37 weeks), birth weight (in grams), rate of neonatal intensive care unit (NICU) admission, gestational age at time of delivery (in weeks), rate of 5-minute Apgar score of less than 7, Apgar score at 1 minute, infant head circumference (in centimeters), infant length (in centimeters), and Apgar score at 5 minutes.

### Statistical Analysis

We used the Review Manager (RevMan) software version 5.4 (Cochrane Collaboration). Continuous data were presented as a mean difference and 95% CI, while dichotomous data were presented as risk ratio (RR) and 95% CI. *P* values were 1-sided, and data were considered significant at *P* < .05. We measured heterogeneity using *I*^2^ and χ^2^ tests. Significant heterogeneity was considered to be present with any χ^2^ or *P* score of less than .10. We used the random-effects model when heterogeneity was found; otherwise, the fixed-effects model was used. We used the technique of excluding 1 study to resolve heterogeneity when applicable. Data were analyzed from August through September 2021.

## Results

### Literature Search

**Figure 1.**
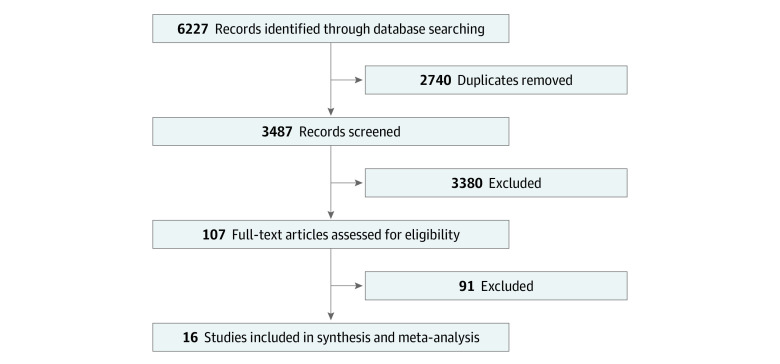
Study Flowchart

Initially, there were 6227 records from the systematic electronic search. After removing duplicates, we were left with 3487 records. There were 107 records suitable for full-text screening after abstract screening. After full-text screening, 16 studies, encompassing 59 138 patients, were ultimately included.^20,21,24,25,26,27,28,29,30,31,32,33,34,35,36,37^ Figure 1 shows the flowchart of this workflow.

### Characteristics of the Included Studies

The included studies were all cohort studies. Details about included studies’ summaries and baseline characteristics are presented in Table 1 and Table 2 and eTable 1 in the Supplement.^20,21,24,25,26,27,28,29,30,31,32,33,34,35,36,37^ Analyzed studies included 14 studies conducted in the United States,^20,21,24,25,26,29,30,31,32,33,34,35,36,37^ 1 study conducted in Canada,^27^ and 1 study conducted in Jamaica.^28^ Study group sizes ranged from 30 individuals who used marijuana vs 25 individuals who did not in Hayes et al^28^ to 11 178 individuals with no marijuana use vs 1245 individuals who used marijuana in Linn et al^29^ (Table 1 and Table 2). Among individuals using marijuana, mean (SD) maternal age ranged from 18.5 (1.8) years in Rodriguez et al^21^ to 29.0 (6.1) years in Conner at al^26^ (for marijuana use ≥10 weeks’ gestation); among individuals not using marijuana, mean (SD) maternal age ranged from 18.8 (1.5) years in Rodriguez et al^21^ to 30.9 (5.8) years in Hoffman et al^20^ (Table 2).

**Table 1.** Summary of Included Studies

Source	Study design	Country	Study groups and sample
Bailey et al,^24^ 2020	Cohort study	United States	Newborns exposed to marijuana: n = 531; control group: n = 531
Conner et al,^25^ 2015	Cohort study	United States	Marijuana use: n = 680; no marijuana use: n = 7458
Conner et al,^26^ 2016	Cohort study	United States	Marijuana use: n = 76; no marijuana use: n = 115
Fried et al,^27^ 1984	Cohort study	Canada	Irregular marijuana use: n = 48; moderate use: n = 18; heavy use: n = 18; no marijuana use: n = 499
Hayes et al,^28^ 1988	Cohort study	Jamaica	Irregular marijuana users: n = 11; moderate use: n = 11; heavy use: n = 8; no marijuana use: n = 25
Hoffman et al,^20^ 2019	Cohort study	United States	Marijuana only at conception: n = 26; marijuana at <10 wk gestation: n = 13; marijuana at ≥10 wk gestation: n = 25; no marijuana use: n = 98
Linn et al,^29^ 1983	Cohort study	United States	No marijuana use: n = 11 178; occasional use: n = 880; weekly use: n = 228; daily use: n = 137
Mark et al,^30^ 2015	Cohort study	United States	Marijuana negative: n = 280; marijuana positive: n = 116
Metz et al,^31^ 2017	Cohort study	United States	Marijuana use: n = 48; no marijuana use: n = 1562
Rodriguez et al,^21^ 2019	Cohort study	United States	Marijuana use: n = 211; no marijuana use: n = 995
Shiono et al,^32^ 1995	Cohort study	United States	Marijuana use: n = 822; no marijuana use: n = 6648
Stein et al,^33^ 2019	Cohort study	United States	Marijuana use: n = 430; no marijuana use: n = 4154
Straub et al,^34^ 2019	Cohort study	United States	Marijuana negative: n = 4075; marijuana positive: n = 1268
Warshak et al,^35^ 2015	Cohort study	United States	Marijuana use: n = 361; no marijuana use: n = 6107
Witter et al,^36^ 1990	Cohort study	United States	Marijuana use: n = 417; no marijuana use: n = 7933
Zuckerman et al,^37^ 1989	Cohort study	United States	Marijuana use: n = 202; no marijuana use: n = 895

**Table 2.** Baseline Characteristic of Included Studies

Source	Study group	Participants, No.	Maternal age, mean (SD), y	Parity, %	Alcohol use, %
Bailey et al,^24^ 2020	Not marijuana exposed	531	24.4 (5.1)	Mean (SD): 1.0 (1.1)	66.40
	Marijuana exposed	531	24.4 (5.3)	Mean (SD): 1.1 (1.2)	66.40
Conner et al,^25^ 2015	Marijuana use	680	24.0 (5.3)	Nulliparity: 33.3	7.60
	Nonuse	7458	25.0 (6.1)	Nulliparity: 37.3	0.80
Conner et al,^26^ 2016	Marijuana use	76	26.4 (4.24)	NA	NA
	Nonuse	115	26.6 (3.87)	NA	NA
Fried et al,^27^ 1984	Nonuse	499	29.3 (NA)	0.33	3
	Irregular use	48	26 (NA)	0.5	2
	Moderate use	18	26.4 (NA)	0.7	11
	Heavy use	18	25.9 (NA)	0.68	10.50
Hayes et al,^28^ 1988	Nonuse	25	NA	NA	NA
	Irregular use	11	NA	NA	NA
	Moderate use	11	NA	NA	NA
	Heavy use	8	NA	NA	NA
Hoffman et al,^20^ 2019	No marijuana use	26	30.9 (5.8)	NA	0
	Marijuana only at conception	13	27.9 (5.7)	NA	65
	Marijuana at <10 wk gestation	25	26.9 (5.9)	NA	12
	Marijuana at ≥10 wk gestation	98	29.0 (6.1)	NA	96
Linn et al,^29^ 1983	No marijuana use	11 178	Age ≥26 y, 71.5%	Parity >1: 50.6	21.90
	Occasional use	880	Age ≥26 y, 46.3%	Parity >1: 35.3	28.10
	Weekly use	229	Age ≥26 y, 38.0%	Parity >1: 39.7	37.60
	Daily use	137	Age ≥26 y, 38.0%	Parity >1: 39.4	29.90
Mark et al,^30^ 2015	Marijuana negative	280	23 (5.9)	NA	2.10
	Marijuana positive	116	22.9 (5)	NA	6.90
Metz et al,^31^ 2017	Marijuana use	48	Age 18-34 y, 89.6%	NA	NR
	Nonuse	1562	Age 18-34 y,, 83.4%	NA	NR
Rodriguez et al,^21^ 2019	Not marijuana exposed	211	18.8 (1.5)	NA	0
	Marijuana exposed	955	18.5 (1.8)	NA	0.40
Shiono et al,^32^ 1995	Not marijuana exposed	822	NA	NA	4.30
	Marijuana exposed	6648	NA	NA	1.30
Stein et al,^33^ 2019	Not marijuana exposed	430	NA	Parity >1: 59.7	NA
	Marijuana exposed	4154	NA	Parity >1: 53.4	NA
Straub et al,^34^ 2019	Marijuana negative	4075	27.04 (5.72)	Nulliparity: 35.63	26.72
	Marijuana positive	1268	25.85 (5.28)	Nulliparity: 38.91	28.08
Warshak et al,^35^ 2015	Marijuana use	361	25.3 (5.9)	NA	NA
	Nonuse	6107	24 (5.2)	NA	NA
Witter et al,^36^ 1990	Marijuana use	417	NA	NA	NA
	Nonuse	7933	NA	NA	NA
Zuckerman et al,^37^ 1989	Marijuana use	202	NA	NA	NA
	Nonuse	895	NA	NA	NA

Abbreviation: NA, not available.

### Quality Assessment

The score for the included studies was between 11.5 and 13.5 out of 14. Most included studies did not examine different levels of exposure (including differences in frequency of use or dosage) associated with the outcome (12 studies [75.0%]). Additionally, most studies did not assess the exposure more than once (14 studies [87.5%]) and did not blind outcome assessors to the exposure status of patients (15 studies [90.8%]). Other quality-associated questions were mainly answered as yes. For example, the research question or objective was clearly stated for all studies and the participation rate of eligible individuals was at least 50% for 15 studies. Full details of the quality assessment are presented in the eTable 2 in the Supplement.

### Outcomes

**Figure 2.**
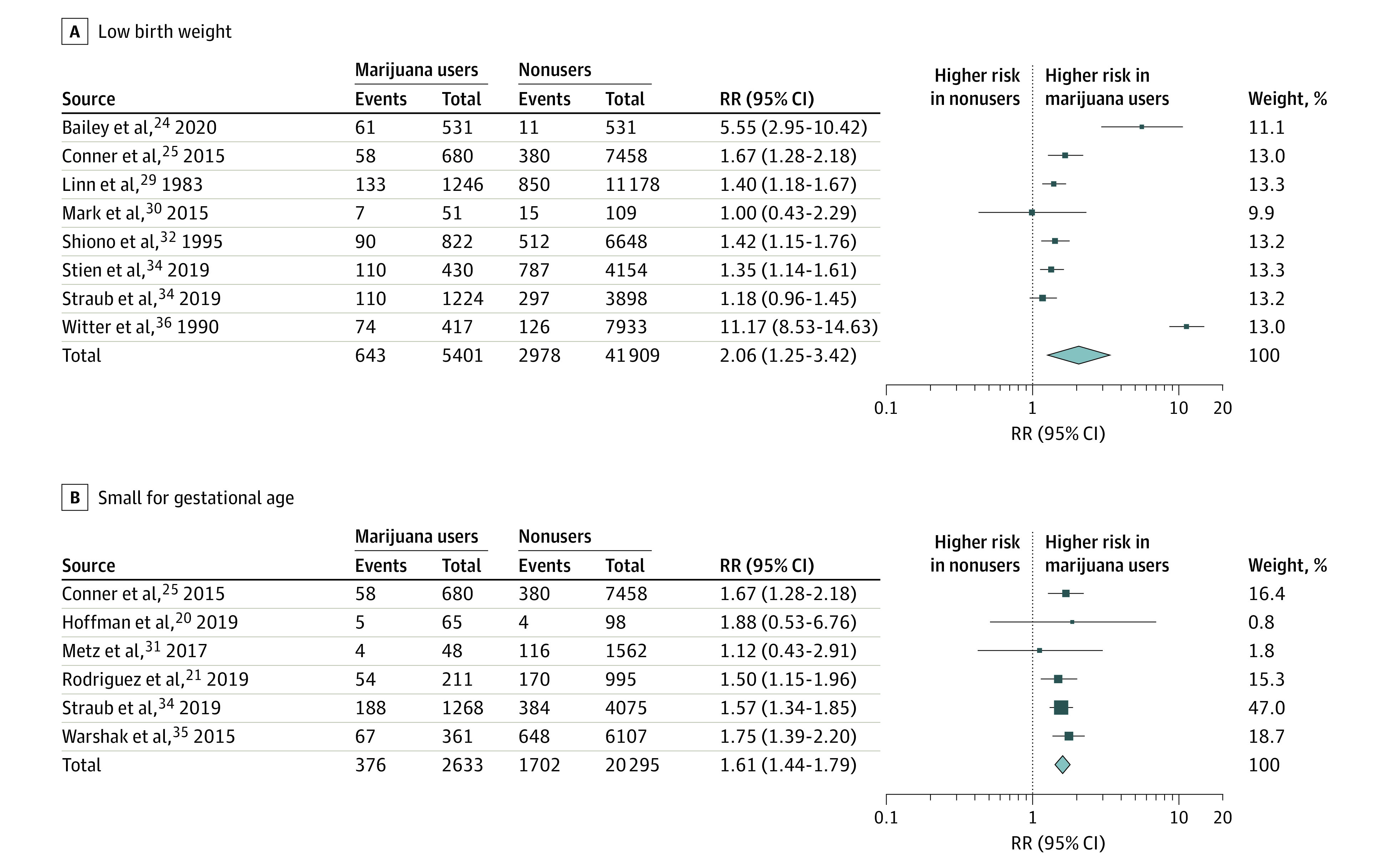
Risk of Low Birth Weight and Small for Gestational Age

**Figure 3.**
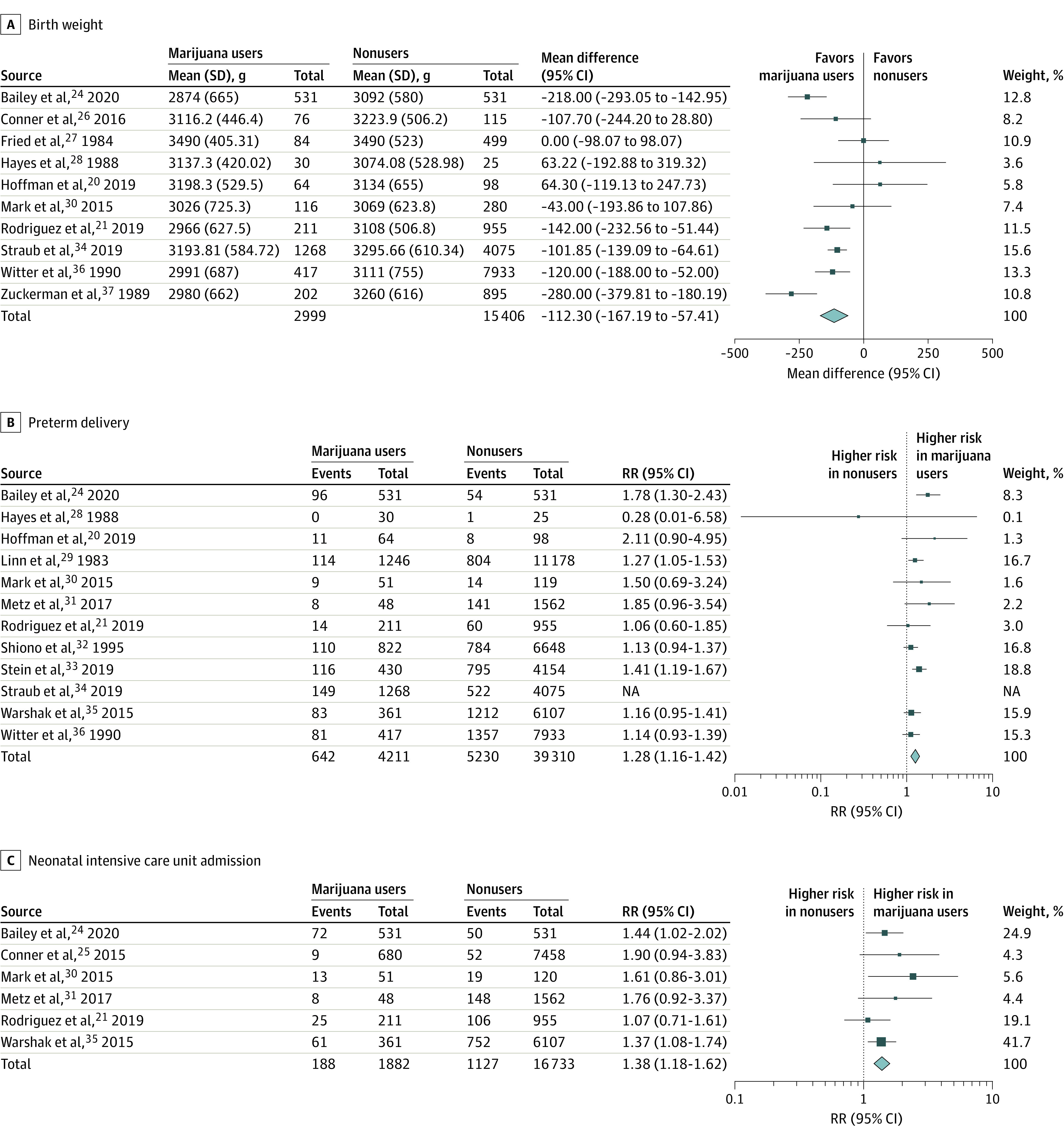
Mean Birth Weight and Risk of Preterm Delivery and Neonatal Intensive Care Unit Admission NA indicates not applicable.

In 8 studies,^24,25,29,30,32,33,34,36^ data on incidence of low birth weight (defined as <2500 g) were reported, with a total of 47 310 included patients. Risk of low birth weight was significantly increased among pregnant women who were exposed vs women who were not exposed to marijuana (RR, 2.06 [95% CI, 1.25 to 3.42]; *P* = .005), but the results were heterogeneous (τ^2^ = 0.49; χ^2^_7_ = 230.25; *P* <.001; *I*^2^ = 97.0%) (Figure 2A).^24,25,29,30,32,34,36^ We could not solve the heterogeneity. When considering a diagnosis of small for gestational age, (defined <fifth percentile by birth weight), 6 studies^20,21,25,31,34,35^ had enough data to be included, with a total of 22 928 patients. There was a significantly increased risk of small for gestational age among pregnant women exposed to marijuana compared with pregnant women who were not exposed (RR, 1.61 [95% CI, 1.44 to 1.79]; P < .001), and the results were homogenous (χ^2^_5_ = 1.56; *P* = .91; *I*^2^ = 0%) (Figure 2B).^20,21,25,31,34,35^ When comparing actual birth weight in grams, 10 studies^20,21,24,26,27,28,30,34,36,37^ had enough data for inclusion, with a total of 18 405 patients. Fetal weight was significantly increased among pregnant women who were not exposed compared with pregnant women exposed to marijuana (mean difference, −112.30 [95% CI, −167.19 to −57.41] g; *P* < .001). The results, however, were heterogeneous (τ^2^ = 4673.87; χ^2^_9_ = 30.18; *P* < .001; *I*^2^ = 70.0%) and we could not solve the heterogeneity (Figure 3A).^20,21,24,25,27,28,30,34,36,37^ There were 3 studies^20,21,37^ with enough data to compare neonatal head circumference, with a total of 2425 patients. Neonatal head circumference was significantly increased among pregnant women who were not exposed compared with pregnant women exposed to marijuana (mean difference, −0.52 [95% CI, −0.95 to −0.09] cm; *P* = .02). However, the results were heterogeneous; the mean difference for individuals using marijuana vs those not using marijuana was −0.10 (95% CI, −0.77 to 0.57) cm for Hoffman et al,^20^ −0.40 (95% CI, −0.72 to −0.08) cm for Rodriguez et al,^21^ and not estimable for Zuckerman et al^37^ (τ^2^ = 0.10; χ^2^_2_ = 6.68; *P* = .04; *I*^2^ = 70.0%) (eFigure 1 in the Supplement). To resolve the heterogeneity, we excluded Zuckerman et al,^37^ which included 1328 patients, and resolved the heterogeneity (τ^2^ = 0.00; χ^2^_9_ = 0.64; *P* = .42; *I*^2^ = 0%). After this exclusion, there was still a significant decrease in mean neonatal head circumference among women with marijuana exposure (mean difference, −0.34 [95% CI, −0.63 to −0.06] cm; *P* = .04) (eFigure 1 in the Supplement). There were sufficient data on the outcome of infant length for 4 studies,^20,21,28,37^ with a total of 2480 patients. There was no significant difference between pregnant women who were not exposed to marijuana and pregnant women who were exposed to marijuana (mean difference, −0.23 [95% CI, −1.26 to 0.81] cm; *P* = .64). However, the results were heterogeneous; the mean difference for individuals using marijuana vs those not using marijuana was 0.60 (95% CI, −0.45 to 1.65) cm for Hayes et al,^28^ 0.70 (95% CI, −0.60 to 2.00) cm for Hoffman et al,^20^ −0.30 (95% CI, −0.89 to 0.29) cm for Rodriquez et al,^21^ and not estimable for Zuckerman et al^37^ (τ^2^ = 0.91; χ^2^_3_ = 21.19; *P* < .001; *I*^2^ = 86.0%) (eFigure 2 in the Supplement). To resolve the heterogeneity, we excluded Zuckerman et al.^37^ This resolved the heterogeneity (τ^2^ = 0.16; χ^2^_2_ = 3.37; *P* = .19; *I*^2^ = 41.0%), but there was still no significant difference between women who were not exposed and those who were exposed (mean difference, 0.17 [95% CI, −0.53 to 0.86] cm; *P* = .02) (eFigure 2 in the Supplement).

There were data on rates of preterm delivery (ie, <37 weeks) for 12 studies,^20,21,24,28,29,30,31,32,33,34,35,36^ totaling 48 864 patients. The results showed a significant increase in preterm delivery among women exposed to marijuana during pregnancy vs no exposure (RR, 1.24 [95% CI, 1.09-1.40]; *P* = .001), but the results were heterogeneous ( τ^2^ = 0.02; χ^2^_11_ =  25.06; *P* = .009; *I*^2^ = 56.0%) (Figure 3B).^20,29,21,24,28,31,32,33,34,35,36^ We resolved heterogeneity by excluding Straub et al,^34^ which included 43 521 patients (τ^2^ = 0.01; χ^2^_10_ = 13.51; *P* = .20; *I*^2^ = 26.0%). The results then continued to show a significant increase in risk of preterm delivery among pregnant women exposed to marijuana (RR, 1.28 [95% CI, 1.16-1.42]; *P* < .001) (Figure 3B).

There were 6 studies^21,24,25,30,31,35^ with data on rates of NICU admission, with a total of 18 615 patients. We found a significantly decreased risk among pregnant women who were not exposed compared with pregnant women who were exposed to marijuana (RR, 1.38 [95% CI, 1.18-1.62]; *P* < .001), and the results were homogenous (χ^2^_5_ = 3.12; *P* = 0.68; *I*^2^ =  0%) (Figure 3C).^21,24,25,30,31,35^

There were 8 studies^20,21,24,27,28,30,34,37^ with data on gestational age at time of delivery (in weeks), with a total of 9864 patients. Although there was a significant difference in risk of preterm births when considering whether the birth occurred before or after 37 weeks and 0 days, there were no significant difference between pregnant women who were not exposed and pregnant women who were exposed to marijuana for mean gestational age at time of delivery (mean difference, −0.03 [95% CI, −0.32 to 0.26] weeks; *P* = .94). These results, however, were heterogeneous; the mean difference for individuals using marijuana vs those not using marijuana ranged from −0.70 (95% CI, −1.02 to 0.57) weeks for Bailey et al^24^ to 0.56 (95% CI, 0.04 to 1.08) weeks for Mark et al^30^ (τ^2^ = 0.12; χ^2^_7_ =  30.63; *P* < .001; *I*^2^ =  77.0%), and we could not solve the heterogeneity (eFigure 3 in the Supplement).

There were enough data in 2 studies^24,26^ to compare Apgar scores at the 1-minute mark, with a total of 1253 patients. The mean Apgar score at 1 minute was significantly decreased among pregnant women who were exposed compared with those who were not exposed to marijuana (mean difference, −0.26 [95% CI, −0.43 to −0.09]; *P* = .002). The results were homogenous; the mean difference for individuals using marijuana vs those not using marijuana was −0.30 (95% CI, −0.49 to −0.11) for Bailey et al^24^ and −0.15 (95% CI, −0.48 to 0.18) for Conner et al^26^ (χ^2^_1_ =  0.59; *P* = .44; *I*^2^ = 0%) (eFigure 4 in the Supplement). There were 3 studies^22,20,26^ with data on Apgar scores at the 5-minute mark, with a total of 1415 patients. There was no significant difference between pregnant women who were exposed to marijuana compared with pregnant women who were not exposed to marijuana in mean Apgar score at 5 minutes (mean difference, −0.06 [95% CI, −0.21 to 0.10]; *P* = .73). The results were heterogeneous; the mean difference for individuals using marijuana vs those not using marijuana was not estimable for Bailey et al,^24^ 0.00 (95% CI, −0.14 to 0.14) for Conner et al,^26^ and 0.06 (95% CI, −0.13 to 0.25) for Hoffman et al^20^ (τ^2^ =  0.01; χ^2^_2_ = 6.17; *P* = .05; *I*^2^ = 68.0%) (eFigure 5 in the Supplement). To resolve the heterogeneity, we excluded Bailey et al,^24^ which included 353 patients. This resolved heterogeneity (τ^2^ =  0.00; χ^2^_1_ = 0.24; *P* = .62; *I*^2^ = 0%), but still no significant difference was seen (mean difference, 0.02 [95% CI, −0.09 to 0.13]; *P* = .65) (eFigure 5 in the Supplement). Additionally, 3 studies^21,25,30^ included enough data to compare the rate of occurrence of Apgar scores less than 7 at 5 minutes of life. This included a total of 9740 patients. There was no significant difference between pregnant women who were not exposed and pregnant women who were exposed to marijuana (RR, 0.76 [95% CI, 0.29 to 2.00]; *P* = .41). However the results were heterogeneous; the risk ratio for individuals using marijuana vs those not using marijuana was 1.37 (95% CI, 0.77 to 2.43) for Conner et al,^25^ not estimable for Mark et al,^30^ and 1.23 (95% CI, 0.35 to 2.33) for Rodriguez et al^21^ (τ^2^ =  0.46; χ^2^_2_ = 5.92; *P* = .05; *I*^2^ = 66.0%) (eFigure 6 in the Supplement). For resolving the heterogeneity, we excluded Mark et al,^30^ which included 9344 patients. Although this resolved heterogeneity (τ^2^ = 0.00; χ^2^_1_ = 0.54; *P* = .46; *I*^2^ = 0%), there was still no significant difference between groups (RR, 1.23 [95% CI, 0.75 to 2.00]; *P* = .45) (eFigure 6 in the Supplement).

## Discussion

This meta-analysis found a significant difference in neonatal outcomes of pregnant women with exposure to marijuana compared with pregnant women without exposure, including increased risk of low birth weight (ie, <2500 g), small for gestational age diagnosis, preterm delivery (ie, <37 weeks), and NICU admission and decreased mean birth weight (in grams), Apgar score at 1 minute, and infant head circumference (in centimeters). No significant differences were found in the outcomes of mean gestational age (in weeks), risk of 5-minute Apgar scores less than 7, mean Apgar score at 5 minutes, or mean infant length (in centimeters).

In an April 2016 meta-analysis, Gunn et al^19^ reported a decrease in birth weight among infants exposed to cannabis products during the fetal period compared with those not exposed, which agrees with our findings. In contrast to our findings, an October 2016 meta-analysis from Conner et al^7^ reported that marijuana use during pregnancy was associated with an increased risk of low birth weight and preterm delivery, but these associations were no longer present when controlling for tobacco use and other confounding factors. Since that time, data have been published that now afford us robust enough numbers to confidently exclude tobacco as a confounding factor, which is also in line with the findings of Haight et al.^38^ In their 2017 cross-sectional study, they found that the frequency of cannabis use was associated with low birth weight delivery, apart from cigarette use.^38^ This is a particularly meaningful finding given that the focus of Haight et al^38^ was to investigate an association between cannabis use during pregnancy and tobacco use, and cannabis use was associated with tobacco use. For clarification, these data speak to the exclusion of concomitant tobacco as a confounding factor in adverse neonatal outcomes. However, at this time, there are no data to differentiate smoking itself (ie, inhalation of marijuana smoke) vs ingestion of the cannabinoids as the main factor associated with an increase in adverse events, to our knowledge.

Cannabinoid receptors, as well as their endogenous ligands, are detected very early in embryonic development.^39^ Additionally, the endocannabinoid system appears to have important roles during these early stages associated with neuronal development and cell survival.^40,41^ These assumptions suggest the hypothesis that fetal exposure to cannabis could be associated with abnormalities in fetal growth and changes in birth outcomes, although no study has found a direct link to date.^42^ Other studies have also proposed that a different mechanism of action, cannabis association with regulation of glucose and insulin, could also act as a teratogen associated with fetal growth.^43^

A recent study provided a third proposed mechanism of action on the fetus associated with endocrine changes in the placenta. Maia et al in 2020^44^ reported that the main psychoactive compound in marijuana, Δ9-tetrahydrocannabinol, disturbs the placental endocrine function as it augments ESR1 and CYP19A1 gene transcription, thus increasing the production of estradiol. Further evidence for this is that cannabinoid and estrogen receptors seem to have overlapping molecular pathways, as was shown by Dobovišek et al in 2016.^45^

We recommend future research to evaluate the maternal outcomes and neonatal outcomes associated with marijuana exposure. Moreover, we recommend assessing the association between marijuana use and other confounders, such as smoking. We also encourage increasing the awareness among women at reproductive age, especially those already pregnant, of the possibility of adverse outcomes associated with marijuana use during pregnancy.

### Limitations

This study has several limitations, including that the analyzed studies were all cohort studies, so they may be liable to bias associated with their retrospective nature. Patient honesty may have also played a role in the quality of this analysis, given that patient truthfulness may be questionable and that most included studies relied at least partially on patients admitting use of marijuana in pregnancy. In addition, many studies did not differentiate levels of marijuana use, in some cases grouping heavy daily users with mothers who may have experimented with marijuana use in pregnancy. Additionally, no studies differentiated between smoking marijuana and other forms of marijuana ingestion; the possibility that some of the outcomes could be partially associated with smoke inhalation, and not necessarily the ingestion of marijuana, is a consideration.

## Conclusions

We found that women using marijuana during their pregnancies were at significantly increased risk of adverse neonatal outcomes, such as low birth weight, preterm delivery, NICU admission, and decreased Apgar score in some situations. Given increasing marijuana legalization and use worldwide, raising awareness and educating patients about these adverse outcomes may help to improve neonatal health.

## Notes

Supplement. **eTable 1.** Summary of Included Studies **eTable 2.** Risk of Bias Assessment of Included Studies **eFigure 1.** Infant Head Circumference **eFigure 2.** Infant Length in Centimeters **eFigure 3.** Gestational Age at Delivery in Weeks **eFigure 4.** Apgar Score at 1 Minute **eFigure 5.** Apgar Score at 5 Minutes **eFigure 6.** Rate of 5 Minute Apgar Scores <7
