# Supplementary material for: Birth Outcomes of Neonates Exposed to Marijuana in Utero: A Systematic Review and Meta-analysis
Source: JAMA Netw Open. 2022 Jan 27;5(1):e2145653. doi: 10.1001/jamanetworkopen.2021.45653 (PMC8796018; doi:10.1001/jamanetworkopen.2021.45653)
Supplement: Supplement. — eTable 1. Summary of Included Studies eTable 2. Risk of Bias Assessment of Included Studies eFigure 1. Infant Head Circumference eFigure 2. Infant Length in Centimeters eFigure 3. Gestational Age at Delivery in Weeks eFigure 4. Apgar Score at 1 Minute eFigure 5. Apgar Score at 5 Minutes eFigure 6. Rate of 5 Minute Apgar Scores <7 [file jamanetwopen-e2145653-s001.pdf]

Supplemental Online Content

Marchand G, Masoud AT, Govindan M, et al. Birth outcomes of neonates exposed to marijuana in utero: a systematic review and meta-analysis. *JAMA Netw Open*. 2022;5(1):e2145653. doi:10.1001/jamanetworkopen.2021.45653

eTable 1. Summary of Included Studies
eTable 2. Risk of Bias Assessment of Included Studies
eFigure 1. Infant Head Circumference
eFigure 2. Infant Length in Centimeters
eFigure 3. Gestational Age at Delivery in Weeks
eFigure 4. Apgar Score at 1 Minute
eFigure 5. Apgar Score at 5 Minutes
eFigure 6. Rate of 5 Minute Apgar Scores <7

This supplemental material has been provided by the authors to give readers additional information about their work.

© 2022 Marchand G et al. *JAMA Network Open.*

eTable 1. Exposure and Results Summaries of Included Studies

Source	Marijuana exposure details	Results
Bailey et al, 2020	"In the Appalachian cohort, 336 marijuana-using women were identified after elimination of those who gave birth to twins and higher order multiples (n = 606). For nine of these an exact match was not available, reducing the final sample to 323 marijuana users and 323 controls"	"Marijuana exposure in utero predicted newborn factors linked to longer-term health and development issues. Effects were not attributable to other comorbidities in this study due to rigorous matching and biochemical verification of marijuana and other drug use. Findings add to growing evidence linking marijuana exposure to adverse birth and longer-term outcomes. Women should be encouraged to avoid marijuana use during pregnancy"
Conner et al, 2015	"Marijuana use was defined as women who used marijuana at least once during pregnancy and were identified through self-report or positive urine drug screen. Two groups were defined, women who used marijuana during pregnancy, and those who did not"	"Marijuana use is common in pregnancy but may not be an independent risk factor for poor neonatal outcomes in term pregnancies"
Conner et al, 2017	"The women received all of their prenatal and substance abuse treatment in an integrated medical and behavioral health program. Women were seen at least twice per month, and urine toxicology testing was completed at every visit"	"Preliminary results indicate that marijuana exposure in the third trimester does not complicate the pregnancy or the delivery process. However, the severity of the infant withdrawal syndrome in the immediate postnatal period may be impacted by marijuana exposure. Because previous study of prenatal marijuana exposure has yielded mixed results, further analysis is needed to determine whether these findings are indeed significant"
Fried et al, 1984	"Infants in singleton births of 583 mothers-to-be who volunteered to participate after becoming informed of the study either by their obstetrician or by notices in the public media"	"Compared to nonuse, an average use of marijuana six or more times per week during pregnancy was associated with a statistically significant reduction of 0.8 weeks in the length of gestation after consideration of the effects of nicotine, alcohol, parity, mother's prepregnancy weight, and the sex of the infant. With similar adjustments no reduction in birth weight was noted. Among the heavy marijuana users, the effect on gestation length was dose dependent"
Hayes et al, 1988	"Ganja consumption patterns prior to pregnancy and during the prenatal and postpartum periods were established by: (a) life history interviews determining the level and nature of ganja use before becoming pregnant; (b) self reports of the level and nature of ganja use during prenatal, interpartum, and postpartum periods; and (c) direct observations and confirmation of key informants in various communities"	"Cluster scores on Brazelton Neonatal Behavior Assessment Scales are analyzed for babies whose mothers used marijuana during pregnancy and compared to a control group. Environmental variables explained significant differences in BNBAS scores"
Hoffman et al, 2019	"Women were enrolled from a public safety-net prenatal clinic at 14-16 weeks gestation from July 2013 until July 2016. Gestational age was timed from the last menstrual period and by ultrasound"	"Prenatal marijuana use adversely affects fetal brain development and subsequent behavioral self-regulation, a precursor to later, more serious problems in childhood. Stopping marijuana use before 10 weeks gestational age prevented these effects. Many mothers refuse to cease use because of familiarity with marijuana and belief in its safety. Higher maternal choline mitigates some of marijuana's adverse effects on the fetus"
Linn et al, 1983	"Women were interviewed following delivery but during the delivery admission. They were asked whether they used marijuana during pregnancy. If so, they were asked whether on the average they used the drug occasionally, weekly, or daily. They did not obtain more detailed information about patterns of marijuana usage during pregnancy. Other data collected included demographic characteristics, other habits and exposures, previous medical and obstetric history, and information on the current pregnancy and its outcome"	"More data are needed to establish firmly or rule out an association between marijuana usage and major malformations. Until more information is available, women should be advised not to use marijuana during pregnancy"
Mark et al, 2015	"The primary exposure was marijuana use, defined either by self-report or detected on urine toxicology testing at the first obstetrical visit. The clinic where the study was performed universally screens all patients initiating prenatal care by both written instrument and urine toxicology after patient consent. Continued use was evaluated serially by urine toxicology results each trimester and at the time of delivery. It is a hospital policy to perform urine toxicology testing on all mothers in labor"	"Prenatal care utilization was equal between marijuana users and non-users. Although marijuana is common among obstetric patients at prenatal care initiation, most cease use by delivery. Marijuana is strongly correlated with cigarette use. We found no differences in birth outcomes or utilization of prenatal care by marijuana exposure"
Metz et al, 2017	"Women with non-anomalous singleton live births ≥24 weeks were considered for inclusion. Women with missing obstetrical history data, and/or drug or tobacco use were excluded. The adverse pregnancy outcome was a composite of SGA, SPTB and HTN. SGA was defined as a birthweight less than the 10 percentile for gestational age.11 SPTB was defined as a preterm delivery (<37 weeks) resulting from spontaneous preterm labor with or without intact membranes"	"Maternal marijuana use was not associated with a composite of SGA, SPTB, or HTN. However, it was associated with an increased risk of neonatal morbidity"
Rodriguez et al, 2019	"Women who receive prenatal care in the maternity program for young women are included in a prospectively collected database.17 For this study, the included mothers were identified by matching women in the clinic database with a list of all women who delivered at University of Colorado Hospital over the same time period. Only women who received prenatal care in the maternity program for young women and ultimately delivered at University of Colorado Hospital were included in order to ensure that data for the primary composite outcome would be available for all women in the cohort"	"In a population of young women with nearly universal biological sampling, marijuana exposure was associated with adverse pregnancy outcomes. The heterogeneity of findings in existing studies evaluating the impact of marijuana on mothers and neonates may result from the incomplete ascertainment of exposure"
Shiono et al, 1995	"Women were interviewed at 23 to 26 weeks' gestation about a variety of risk factors thought to be associated with adverse pregnancy outcomes. Women were excluded if they had one of the following: diabetes requiring insulin, hypertension or heart disease requiring medication, chronic renal disease, multiple pregnancy, Rh sensitization, current use of corticosteroids, history of cervical incompetence or cerclage, receipt of antibiotics in the past 2 weeks, use of tocolytic agents before enrollment in the study, and intent to deliver at a nonstudy hospital"	"In this population of women receiving prenatal care, cocaine use was uncommon and was not related to most adverse birth outcomes. Marijuana use was relatively common and was not related to adverse pregnancy outcomes. Tobacco is still the most commonly abused drug during pregnancy, 15% of all cases of low birth weight in this study could have been prevented if women did not smoke cigarettes during pregnancy"
Stein et al, 2019	"Subjects were considered opioid exposed if they had a BSAS treatment record for a primary substance use of heroin, oxycodone, and other opiates in the year preceding delivery"	"Findings conclude that there is a relationship between these 2 categories of substances, whether biochemical or socioeconomic, and justifies the need for further study. We also examined the demographics of a large group of women to evaluate the presence of other high-risk characteristics associated with concomitant opioid and marijuana use in pregnancy"

Straub et al, 2019	"A woman was considered a "marijuana user" if she had a positive UDS for marijuana any time during her pregnancy. A woman for whom all UDS results during pregnancy and/or at delivery were negative was considered a "nonmarijuana user." We chose this objective documentation of marijuana use due to prior studies showing poor correlation between self-reported maternal substance use ("reported use") and drug screens, and poor correlation between reported use and documentation in the electronic record. The study was reviewed and approved by the local institutional review board"	"Marijuana exposure verified by UDS was associated with LBW and SGA. However, recreational marijuana legalization and availability did not have a direct impact on newborns' risk of LBW or SGA"
Warshak et al, 2015	"Marijuana users were designated as such if they reported use during the course of their prenatal care or at the time of delivery, or if at any point during the pregnancy they had a positive toxicology screen for tetrahydrocannabinol. Universal drug screening was not used during the study period but was performed in pregnancies deemed to be high risk for substance abuse, secondary to known history of substance abuse, poor prenatal care or social/medical risk factors for drug abuse. The entire medical record was reviewed, and if prenatal records, laboratory results and the inpatient records revealed no evidence of use, patients were classified as nonusers"	"Maternal marijuana use does not increase the risk of adverse obstetrical outcomes or fetal anomalies but does increase the risk for small for gestational age and neonatal intensive care unit admission"
Witter et al, 1990	"A retrospective analysis of our data from 1983 through 1985 inclusive was conducted. Of 8350 records searched, 417 patients were identified who used marijuana but no other illicit drugs. The pregnancy outcomes of these patients were compared with the remaining 7933 patients who comprised the rest of the population"	"This report summarizes our experience over a 3-y period with marijuana use in pregnancy"
Zuckerman et al, 1989	"The marijuana users were identified through self-report and urine analysis"	"We conclude that use of marijuana during pregnancy is associated with impaired fetal growth"

© 2022 Marchand G et al. *JAMA Network Open.*

eTable 2. Risk of Bias Assessment of Included Studies

ID	1	2	3	4	5	6	7	8	9	10	11	12	13	14	Score
Bailey 2020	Yes	Yes	Yes	Yes	Yes	Yes	Yes	No	Yes	No	Yes	No	Yes	Yes	12.5
Conner 2015	Yes	Yes	Yes	Yes	Yes	Yes	Yes	No	Yes	No	Yes	No	Yes	Yes	12.5
Conor 2017	Yes	Yes	Yes	Yes	No	Yes	Yes	No	Yes	No	Yes	No	Yes	No	11.5
Fried 1984	Yes	Yes	Yes	Yes	No	Yes	Yes	Yes	Yes	No	Yes	No	Yes	Yes	12.5
Hayes 1988	Yes	No	No	No	Yes	Yes	Yes	Yes	Yes	No	Yes	No	Yes	Yes	11.5
Hoffman 2019	Yes	Yes	Yes	Yes	No	Yes	Yes	Yes	Yes	Yes	Yes	Yes	Yes	Yes	13.5
Linn 1983	Yes	Yes	Yes	Yes	No	Yes	Yes	No	Yes	No	Yes	No	Yes	Yes	12
Mark 2015	Yes	Yes	Yes	Yes	Yes	Yes	Yes	No	Yes	Yes	Yes	No	Yes	Yes	13
Metz 2017	Yes	Yes	Yes	Yes	Yes	Yes	Yes	No	Yes	No	Yes	No	Yes	Yes	12.5
Rodriguez 2019	Yes	Yes	Yes	Yes	Yes	Yes	Yes	No	Yes	No	Yes	No	Yes	Yes	12.5
Shiono 1995	Yes	Yes	Yes	Yes	No	Yes	Yes	No	Yes	No	Yes	No	Yes	Yes	12
Stein 2019	Yes	Yes	Yes	Yes	Yes	Yes	Yes	No	Yes	No	Yes	No	Yes	Yes	12.5
Straub 2019	Yes	Yes	Yes	Yes	Yes	Yes	Yes	No	Yes	No	Yes	No	Yes	Yes	12.5
Warshak 2015	Yes	Yes	Yes	Yes	Yes	Yes	Yes	No	Yes	No	Yes	No	Yes	Yes	12.5
Witter 1990	Yes	Yes	Yes	Yes	No	Yes	Yes	No	Yes	No	Yes	No	Yes	No	11.5

© 2022 Marchand G et al. *JAMA Network Open.*

Zuckerman 1989	Yes	Yes	Yes	Yes	No	Yes	Yes	Yes	Yes	No	Yes	No	Yes	No	12

1. Was the research question or objective in this paper clearly stated?
2. Was the study population clearly specified and defined?
3. Was the participation rate of eligible persons at least 50%?
4. Were all the subjects selected or recruited from the same or similar populations (including the same time period)? Were inclusion and exclusion criteria for being in the study prespecified and applied uniformly to all participants?
5. Was a sample size justification, power description, or variance and effect estimates provided?
6. For the analyses in this paper, were the exposure(s) of interest measured prior to the outcome(s) being measured?
7. Was the timeframe sufficient so that one could reasonably expect to see an association between exposure and outcome if it existed?
8. For exposures that can vary in amount or level, did the study examine different levels of the exposure as related to the outcome (e.g., categories of exposure, or exposure measured as continuous variable)?
9. Were the exposure measures (independent variables) clearly defined, valid, reliable, and implemented consistently across all study participants?
10. Was the exposure(s) assessed more than once over time?
11. Were the outcome measures (dependent variables) clearly defined, valid, reliable, and implemented consistently across all study participants?
12. Were the outcome assessors blinded to the exposure status of participants?
13. Was loss to follow-up after baseline 20% or less?
14. Were key potential confounding variables measured and adjusted statistically for their impact on the relationship between exposure(s) and outcome(s)?

© 2022 Marchand G et al. *JAMA Network Open.*

	Marijuana users			Non users				Mean Difference
Study or Subgroup	Mean	SD	Total	Mean	SD	Total	Weight	IV, Random, 95% CI
Hoffman 2019	34.4	1.5	64	34.5	2.8	98	18.5%	-0.10 [-0.77, 0.57]
Rodriguez 2019	33.2	2.18	211	33.6	1.85	955	81.5%	-0.40 [-0.72, -0.08]
Zuckerman 1989	33.4	2.3	202	34.3	1.8	895		Not estimable
**Total (95% CI)**			**275**			**1053**	**100.0%**	**-0.34 [-0.63, -0.06]**

Heterogeneity: Tau² = 0.00; Chi² = 0.64, df = 1 (P = 0.42); I² = 0%

Test for overall effect: Z = 2.36 (P = 0.02)

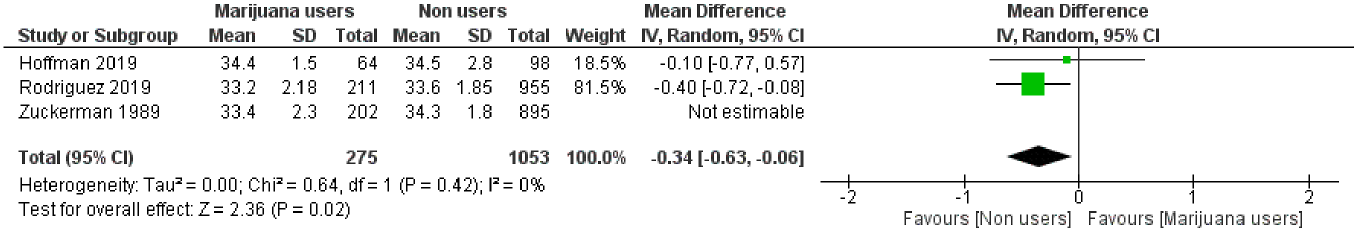

eFigure 1. Infant Head Circumference

© 2022 Marchand G et al. *JAMA Network Open.*

	Marijuana users			Non users				Mean Difference
Study or Subgroup	Mean	SD	Total	Mean	SD	Total	Weight	IV, Random, 95% CI
Hayes 1988	51.3	2.74	30	50.7	0.95	25	28.3%	0.60 [-0.45, 1.65]
Hoffman 2019	49.6	3	64	48.9	5.4	98	21.1%	0.70 [-0.60, 2.00]
Rodriguez 2019	48.5	3.92	211	48.8	4	955	50.6%	-0.30 [-0.89, 0.29]
Zuckerman 1989	48.3	3.5	202	49.8	3	895		Not estimable
**Total (95% CI)**			**305**			**1078**	**100.0%**	**0.17 [-0.53, 0.86]**

Heterogeneity: Tau² = 0.16; Chi² = 3.37, df = 2 (P = 0.19); I² = 41%
Test for overall effect: Z = 0.47 (P = 0.64)

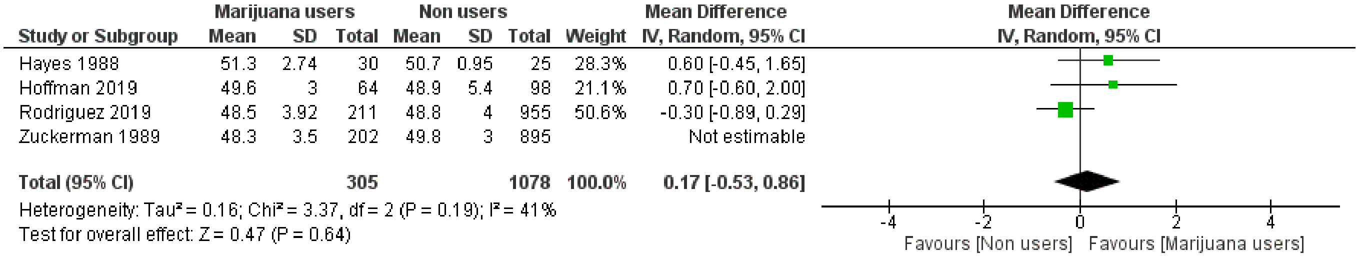

eFigure 2. Infant Length in Centimeters

© 2022 Marchand G et al. *JAMA Network Open.*

Study or Subgroup	Marijuana users			Non users			Weight	Mean Difference
	Mean	SD	Total	Mean	SD	Total		IV, Random, 95% CI
Bailey 2020	38.1	3.1	531	38.8	2.2	531	14.9%	-0.70 [-1.02, -0.38]
Fried 1984	39.9	1.6	84	39.7	1.67	499	14.1%	0.20 [-0.17, 0.57]
Hayes 1988	39.75	0.78	30	39.5	2.1	25	7.0%	0.25 [-0.62, 1.12]
Hoffman 2019	39.2	1.6	64	38.7	2.8	98	9.2%	0.50 [-0.18, 1.18]
Mark 2015	38.86	2.1	116	38.3	3	280	11.6%	0.56 [0.04, 1.08]
Rodriguez 2019	38.7	3.34	211	39.1	2.16	955	12.4%	-0.40 [-0.87, 0.07]
Straub 2019	38.66	2.61	1268	38.61	2.69	4075	17.2%	0.05 [-0.12, 0.22]
Zuckerman 1989	38.9	2.7	202	39.2	1.8	895	13.7%	-0.30 [-0.69, 0.09]
**Total (95% CI)**			**2506**			**7358**	**100.0%**	**-0.03 [-0.32, 0.26]**

Heterogeneity: $Tau^2 = 0.12$; $Chi^2 = 30.63$, df = 7 ($P < 0.0001$); $I^2 = 77\%$
Test for overall effect: Z = 0.20 (P = 0.84)

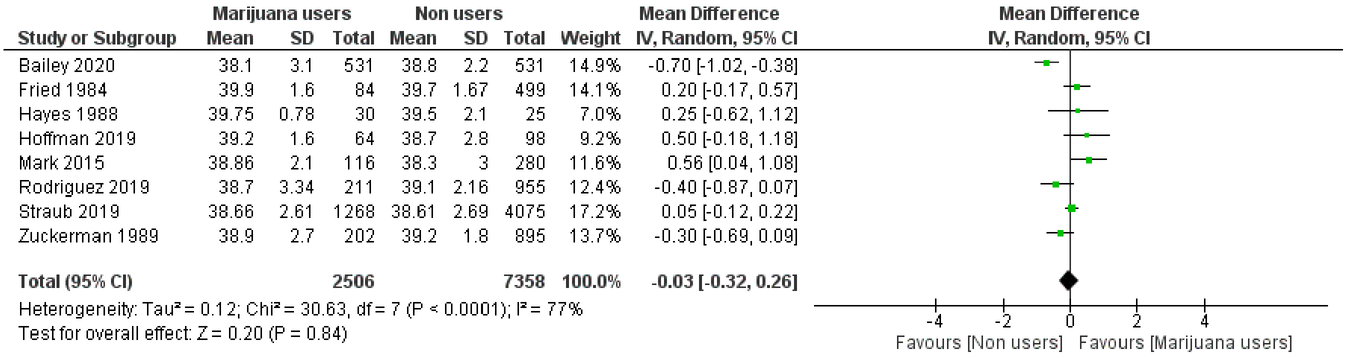

eFigure 3. Gestational Age at Delivery in Weeks

© 2022 Marchand G et al. *JAMA Network Open.*

Study or Subgroup	Marijuana users Mean	SD	Total	Non users Mean	SD	Total	Weight	Mean Difference IV, Fixed, 95% CI
Bailey 2020	7.5	1.8	531	7.8	1.4	531	74.4%	-0.30 [-0.49, -0.11]
Conor 2017	7.89	1.21	76	8.04	1.03	115	25.6%	-0.15 [-0.48, 0.18]
**Total (95% CI)**			**607**			**646**	**100.0%**	**-0.26 [-0.43, -0.09]**

Heterogeneity: Chi² = 0.59, df = 1 (P = 0.44); I² = 0%
Test for overall effect: Z = 3.06 (P = 0.002)

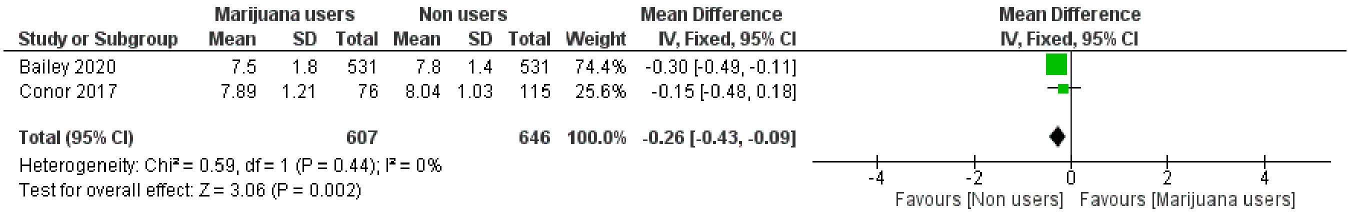

eFigure 4. Apgar Score at 1 Minute

© 2022 Marchand G et al. *JAMA Network Open.*

	Marijuana users			Non users				Mean Difference
**Study or Subgroup**	**Mean**	**SD**	**Total**	**Mean**	**SD**	**Total**	**Weight**	**IV, Random, 95% CI**
Bailey 2020	8.6	1.4	531	8.8	0.8	531		Not estimable
Conor 2017	8.91	0.524	76	8.91	0.388	115	66.6%	0.00 [-0.14, 0.14]
Hoffman 2019	8.8	0.5	64	8.74	0.76	98	33.4%	0.06 [-0.13, 0.25]
**Total (95% CI)**			**140**			**213**	**100.0%**	**0.02 [-0.09, 0.13]**

Heterogeneity: Tau² = 0.00; Chi² = 0.24, df = 1 (P = 0.62); I² = 0%
Test for overall effect: Z = 0.35 (P = 0.73)

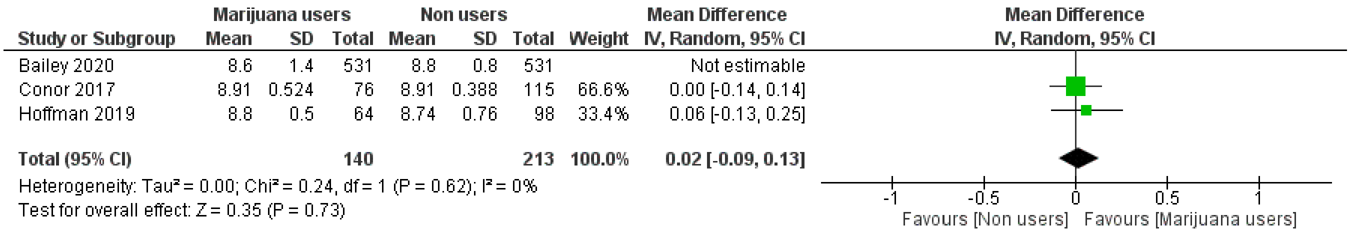

eFigure 5. Apgar Score at 5 Minutes

© 2022 Marchand G et al. *JAMA Network Open.*

	Marijuana users		Non users			Risk Ratio
Study or Subgroup	Events	Total	Events	Total	Weight	M-H, Random, 95% CI
Conner 2015	13	680	104	7458	73.3%	1.37 [0.77, 2.43]
Mark 2015	2	280	5	116		Not estimable
Rodriguez 2019	5	211	26	995	26.7%	0.91 [0.35, 2.33]
**Total (95% CI)**		**891**		**8453**	**100.0%**	**1.23 [0.75, 2.00]**
Total events	18		130			

Heterogeneity: $Tau^2 = 0.00$; $Chi^2 = 0.54$, df = 1 (P = 0.46); $I^2 = 0\%$
Test for overall effect: Z = 0.82 (P = 0.41)

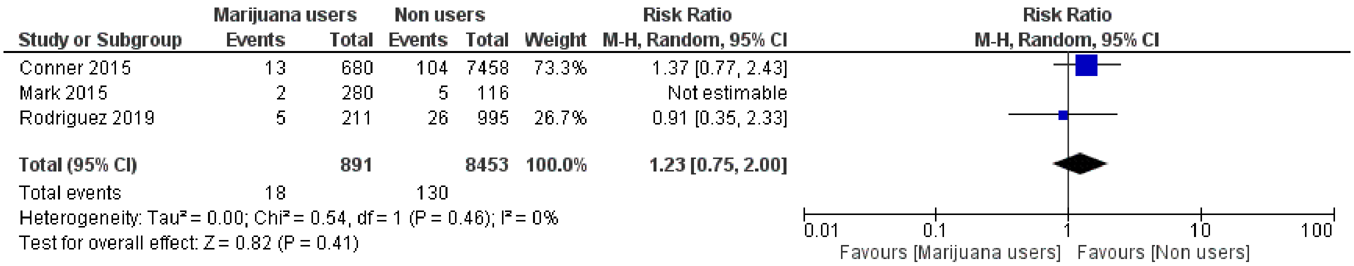

eFigure 6. Rate of 5 Minute Apgar Scores <7

© 2022 Marchand G et al. *JAMA Network Open.*
